# Mesothelioma of the Tunica Vaginalis Testis Initially Treated as Hydrocele: A Case Report

**DOI:** 10.1002/iju5.70257

**Published:** 2026-09-03

**Authors:** Jun‐ichi Hori, Satoshi Yamaguchi, Teppei Ohyama, Hironori Ishida, Yasunari Takakuwa, Masaaki Sato, Akio Homma, Seiji Matsumoto

**Affiliations:** ^1^ Department of Urology Kitasaito Hospital Asahikawa Japan; ^2^ Department of Pathology Sapporo Clinical Laboratory Center Sapporo Japan; ^3^ Department of Urology Moriyama Hospital Asahikawa Japan; ^4^ Headquarters for Research Promotion Asahikawa Medical University Asahikawa Japan

**Keywords:** asbestos, hemiscrotectomy, hydrocele, mesothelioma, tunica vaginalis testis

## Abstract

**Introduction:**

Herein, we report a case of mesothelioma of the tunica vaginalis testis (MTVT), treated as a hydrocele.

**Case Presentation:**

A 65‐year‐old man was referred to our hospital after MTVT was incidentally diagnosed during hydrocelectomy performed for recurrent hydrocele. Left hemiscrotectomy was performed, and pathological examination revealed an epithelioid‐type MTVT with invasion into the testis and spermatic cord. Immunohistochemistry was positive for D2‐40 and calretinin, and negative for cytokeratin 5/6 and carcinoembryonic antigen. The patient remained disease‐free for 12 months postoperatively without adjuvant treatment.

**Conclusion:**

MTVT should be considered in patients with long‐standing or recurrent hydrocele. Wide surgical excision is essential for local control.

## Keynote Message

Here, we report a rare case of mesothelioma of the tunica vaginalis testis that was diagnosed after hydrocelectomy and successfully treated with hemiscrotectomy. The patient remains recurrence‐free after 12 months without adjuvant therapy. A wide surgical excision is essential for effective disease management.

## 1 Introduction

Mesothelioma of the Tunica Vaginalis Testis (MTVT) is a rare, aggressive neoplasm. Murai [1] reported 1786 cases of mesothelioma and found that 1213 cases (68%) were derived from the pleura and only six cases (0.3%) arose from the tunica vaginalis testis. The diagnosis of MTVT is complicated, and there are no guidelines or evidence regarding standard treatment; only a few case series have been published. It arises from the serous lining that covers the testis and epididymis or the spermatic cord and parietal lamina [1, 2]. Asbestos exposure, trauma, recurrent hydrocele, and prolonged inflammation of the testes are recognized risk factors [1, 2, 3]. Prognosis is poor, especially in patients with metastasis‐like, local, and lung lymph node involvement [3]. Herein, we report a case of MTVT diagnosed by tissue sampling after multiple episodes of hydrocele aspiration, followed by radical orchiectomy.

## 2 Case Presentation

**FIGURE 1:**
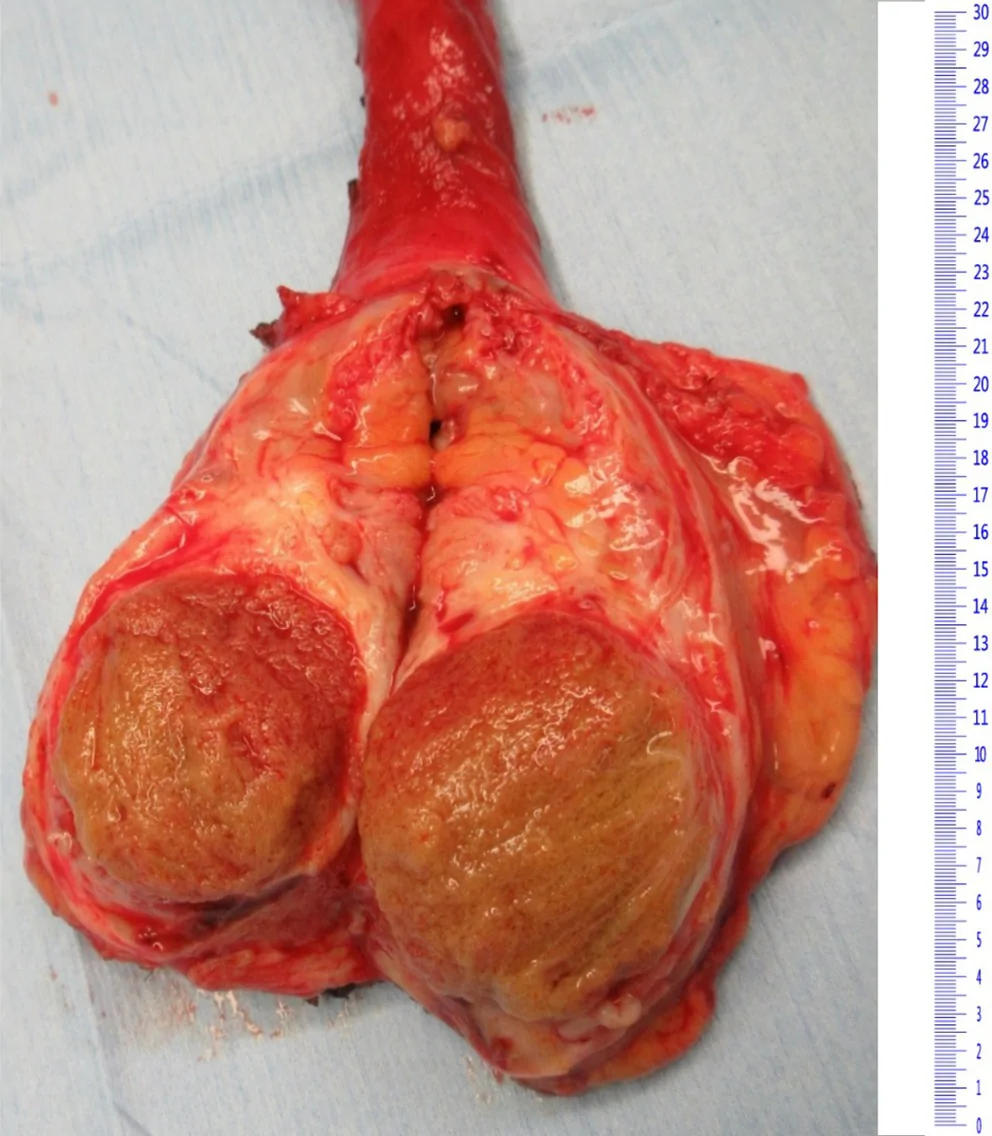
Macroscopic findings showing no gross abnormalities in the testis, epididymis, or hydrocele wall.

**FIGURE 2:**
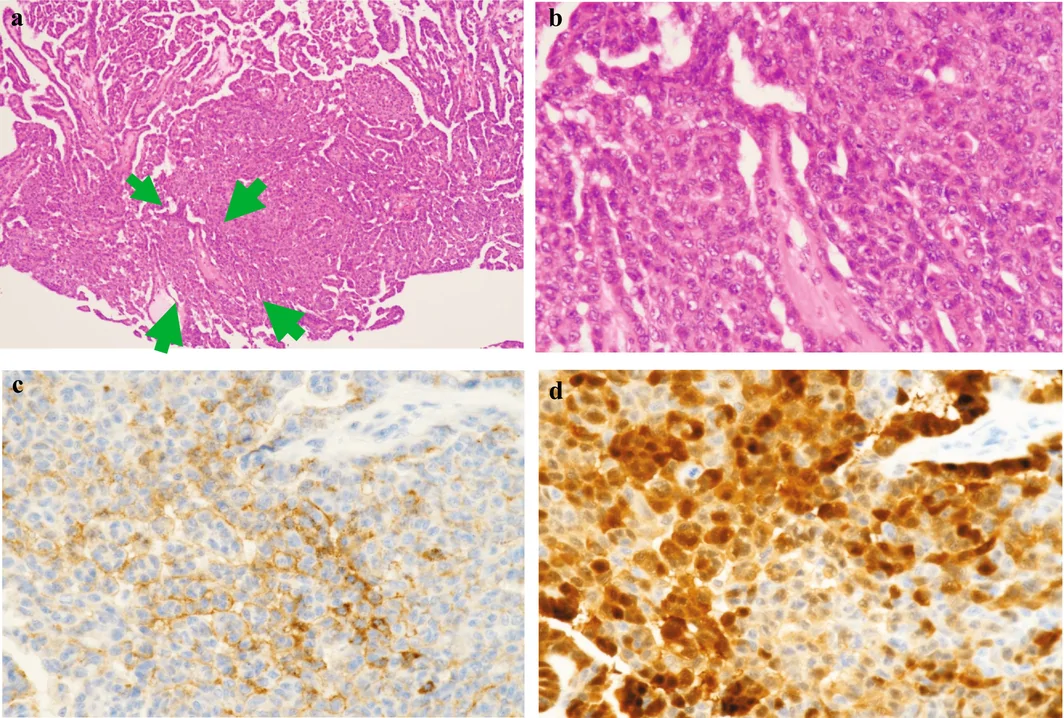
(a) Epithelioid‐type mesothelioma invading the testis, 100×; (b) Epithelioid‐type mesothelioma invading the testis, 400×; (c) Tumor cells positive for D2‐40; (d) Tumor cells positive for calretinin.

A 63‐year‐old man had previously undergone more than 20 aspirations at another hospital for recurrent left‐sided hydrocele testis. He subsequently underwent hydrocelectomy at the same hospital. An unusual nodular thickening was identified in the tunica vaginalis during the operation; therefore, part of it was resected and submitted for histopathological examination, which revealed MTVT. The patient was subsequently referred to our hospital for further treatment. Preoperative CT revealed no metastasis. Left hemiscrotectomy was subsequently performed, and macroscopic analysis revealed no abnormalities in the testes, epididymis, or hydrocele walls (Figure 1). Pathological examination revealed an epithelioid MTVT invading the testes and spermatic cord via lymphovascular spreading (Figure 2a,b). Immunohistochemistry revealed that the tumor cells were positive for D2‐40 and calretinin but negative for cytokeratin 5/6 and carcinoembryonic antigen (Figure 2c,d). The resection margins were negative. A medical oncologist was consulted; given the absence of residual disease, adjuvant chemotherapy was deferred. Surveillance CT scans were planned every 3 months for the first 2 years, then every 6 months for 5 years. The patient did not show any signs of recurrence 12 months postoperatively.

## 3 Discussion

Due to its rarity and the absence of disease‐specific clinical or radiological features, MTVT is difficult to diagnose preoperatively [4]. The present case exemplifies a typical diagnostic pathway in which MTVT was incidentally identified during hydrocelectomy in a patient without asbestos exposure. Although ultrasonography or contrast‐enhanced computed tomography may reveal tunical surface irregularities or soft‐tissue nodules, such findings are reported only in a minority of cases and are often limited by the small size of the lesions [3]. Doppler ultrasonography has shown potential utility; however, its diagnostic reliability remains uncertain due to the paucity of data [3, 5]. Some showed hemorrhagic hydrocele fluid or positive fluid cytology during aspiration being the suspect of MTVT, but in most cases, tumors are identified intraoperatively [1, 5].

Asbestos exposure is a well‐established risk factor for pleural and peritoneal mesothelioma [1, 2, 3, 4, 5]; however, fewer than half of the reported MTVT cases have a documented history of exposure [6]. Plas et al. [7] reported that only 34.2% of patients had prior asbestos exposure, suggesting that additional etiological factors may contribute to tumor development. Chronic inflammatory conditions, including long‐standing hydrocele, epididymitis, orchitis, and prior inguinal surgery, have been proposed as alternative risk factors [1, 2, 3, 4, 5, 6, 7]. Gurdal and Erol [8] first described MTVT arising in the context of a long‐standing hydrocele, and subsequent reports have noted an association with hydrocele in more than half of cases, raising the possibility that chronic serosal irritation may promote malignant transformation.

Histologically, MTVT is classified into epithelioid, biphasic, and sarcomatoid subtypes [3, 4, 5, 6, 7, 8]. Immunohistochemical evaluation plays a critical role in differentiating MTVT from metastatic adenocarcinomas and other paratesticular malignancies. Positive staining for calretinin, WT1, and D2‐40, combined with negative staining for carcinoembryonic antigen and Leu‐M1, supports the diagnosis of mesothelioma [3, 8].

Surgical resection remains the cornerstone of treatment for this condition. Plas et al. [7] reported that more than half of their patients experienced local or distant recurrence within two years of initial therapy, underscoring the importance of hemiscrotectomy with wide resection margins. Surgical excision is the preferred approach for local recurrence [7]. Although systemic chemotherapy with pemetrexed plus cisplatin has demonstrated survival benefits in malignant pleural mesothelioma, its efficacy in MTVT remains unclear because of the rarity of the disease and the lack of prospective data [9]. Several reports have suggested that MTVT is generally unresponsive to chemotherapy and radiotherapy, further emphasizing the need for complete surgical excision [4, 5]. Grogg et al. [10] identified spermatic cord or scrotal invasion as predictors of local recurrence, whereas metastatic progression was associated with tumor size, necrosis, mitotic activity, angiolymphatic invasion, and patient age. They proposed that wide surgical resection within a multimodal framework should be considered, although the optimal role of systemic therapy remains unclear [10].

The present case represents a locally advanced MTVT with invasion into the testis and spermatic cord, but without metastatic disease. Complete resection was achieved via hemiscrotectomy, and the patient remained recurrence‐free for 12 months without adjuvant therapy. Considering the high risk of early recurrence reported in the literature, continued surveillance with scheduled computed tomography is warranted.

## 4 Conclusion

We report a rare case of MTVT incidentally diagnosed during hydrocelectomy and subsequently managed with hemiscrotectomy. Current evidence supports wide surgical excision as an essential component of treatment; however, the role of multimodal therapy remains uncertain. Careful postoperative surveillance is necessary due to the potential for early recurrence.

## Ethics Statement

The authors have nothing to report.

## Consent

Written informed consent was obtained from the patient for the publication of this case report and the accompanying images.

## Conflicts of Interest

The authors declare no conflicts of interest.

## Data Availability

The data that support the findings of this study are available on request from the corresponding author. The data are not publicly available due to privacy and ethical restrictions.
